# The Relationship Between Sense of Coherence and Occupational Burnout Among Psychiatric Nurses: A Cross-Sectional Study in Inpatient Psychiatric Wards in Poland

**DOI:** 10.3390/nursrep15090320

**Published:** 2025-09-04

**Authors:** Kinga Kołodziej, Ewa Wilczek-Rużyczka, Anna Majda

**Affiliations:** 1 Department of Theory and Fundamentals of Nursing, Institute of Nursing and Midwifery Faculty of Health Sciences, Jagiellonian University Collegium Medicum, Michałowskiego 12 St., 31-126 Cracow, Poland; anna.majda@uj.edu.pl; 2 Faculty of Psychology, SWPS University, al. Jana Pawła II 39A, 31-864 Cracow, Poland; ewilczek-ruzyczka@swps.edu.pl

**Keywords:** psychiatric nurses, sense of coherence, burnout

## Abstract

**Background**: Sense of coherence constitutes a significant personal resource that underpins the harmonious professional functioning of nurses employed in psychiatric inpatient wards. It serves as a protective factor, enabling effective coping with the psychophysical burden arising from a demanding and stress-inducing work environment, while also supporting the maintenance of a high level of job satisfaction. Regular assessment of the sense of coherence among psychiatric nursing staff is essential for the early identification of individuals at risk of developing occupational burnout. The aim of the present study was to determine the relationship between the level of sense of coherence and the degree of occupational burnout among nurses working in inpatient psychiatric units. **Methods**: The study employed a cross-sectional design and utilized standardized psychometric instruments, including The Sense of Coherence Questionnaire (SOC-29) to assess the level of coherence, and the Maslach Burnout Inventory (MBI) to measure occupational burnout. Additionally, a self-developed questionnaire was used to collect sociodemographic data. The research was conducted in five psychiatric hospitals in Poland between January and June 2023. The sample consisted of 555 nurses (449 women and 106 men) employed in inpatient psychiatric wards. Statistical analyses included descriptive statistics, Pearson’s correlation coefficients to examine relationships between variables, and multiple linear regression to identify predictors of burnout dimensions. Significance level set at *p* < 0.05. **Results**: The mean global sense of coherence score among psychiatric nurses was 124.68 (SD = 45.81), with manageability scoring highest among subscales (43.83, SD = 16.28). Average occupational burnout scores were emotional exhaustion 28.75 (SD = 16.39), depersonalization 13.55 (SD = 9.71), and reduced personal accomplishment 23.61 (SD = 11.11). Significant negative correlations were found between sense of coherence (and its components) and all burnout dimensions (*p* < 0.001). Manageability was the strongest predictor of lower emotional exhaustion (β = −0.73), depersonalization (β = −0.65), and reduced personal accomplishment (β = −0.65), while meaningfulness predicted depersonalization (β = 0.37, *p* = 0.012). These results indicate that higher sense of coherence, especially manageability, is linked to reduced burnout among psychiatric nurses. **Conclusions**: The study revealed significant negative associations between sense of coherence and all dimensions of occupational burnout, with manageability emerging as the strongest protective factor. Nurses with higher levels of sense of coherence reported lower emotional exhaustion, depersonalization, and reduced personal accomplishment. These findings highlight the importance of incorporating sense of coherence assessment into strategies for identifying individuals at increased risk of burnout.

## 1. Introduction

Personal resources are defined as the internal characteristics of an individual that enable effective coping, particularly in emotionally demanding and stressful situations. They play fundamental role not only in the process of adapting to adverse conditions but also in achieving success in both personal and professional life [1]. Personal resources encompass psychological traits and predispositions that foster psychological resilience and support optimal individual functioning.

Within the framework of Aaron Antonovsky’s salutogenic concept, the overarching factor in coping with stress is the sense of coherence, which shapes the way stressful situations are perceived and interpreted. It comprises three core components: comprehensibility, manageability, and meaningfulness. Comprehensibility refers to the cognitive appraisal of stimuli as structured, predictable, and understandable. Manageability (also referred to as controllability) reflects the belief in possessing adequate resources—both external and internal—necessary to meet situational demands. Meaningfulness, representing the emotional–motivational dimension, reflects the conviction that life and its inherent challenges are worth engagement. Individuals with a high sense of coherence are more likely to engage in actions oriented toward constructive problem-solving [2,3,4,5,6,7].

The salutogenic model focuses on identifying factors that facilitate the maintenance of health despite exposure to stressors, which aligns with the stress theories proposed by Richard Lazarus and Judith Cohen [8] as well as Hans Selye [9]. According to Antonovsky’s perspective, every individual is continuously confronted with a variety of stressors that require adaptation. The preservation of health depends on the ability to mobilize internal resources in such a way that the perception of these stimuli does not disrupt overall psychophysical well-being [2,4].

The concept of “occupational burnout” was introduced into the scientific literature by American psychiatrist Herbert Freudenberger in his 1974 article Staff Burn-Out, in which he described a decline in motivation and engagement, accompanied by psychosomatic symptoms, among volunteers in a drug rehabilitation center [10]. Independently, Christina Maslach investigated occupational stress in service- and care-related professions, focusing on coping mechanisms for emotional demands [11,12,13,14]. These studies led to the development of a model defining burnout as “*a psychological syndrome of emotional exhaustion*, *depersonalization*, *and reduced personal accomplishment that can occur among individuals who work with other people in some capacity*” [15] (p. 99). In later collaboration with Susan Jackson, the third component—reduced personal accomplishment—was incorporated into the model [15].

Emotional exhaustion denotes chronic fatigue stemming from excessive demands, expressed as diminished energy and motivation, irritability, and psychosomatic complaints. Depersonalization refers to the emergence of a detached, cynical attitude toward others—initially a defensive strategy against overinvolvement, but ultimately fostering dehumanizing tendencies. Reduced personal accomplishment involves loss of confidence in one’s competence, diminished job satisfaction, heightened self-criticism, and a sense of meaninglessness, potentially culminating in professional withdrawal [13,14,15].

The development of burnout reflects a complex interplay between individual resources and environmental conditions, with the balance between job demands and emotional, psychological, and organizational resources as a critical determinant. Organizational climate, leadership style, and interpersonal relationships may act either protectively or as risk factors. Contemporary approaches emphasize the dynamic, reciprocal interaction between the individual and the work environment in shaping vulnerability to burnout [16,17,18].

Although occupational burnout affects numerous professional groups, it is particularly prevalent among psychiatric nurses [19,20]. Contributing factors include patient aggression, excessive workload, insufficient supervisory support, staffing shortages, ineffective team communication, and inadequate professional preparation [21,22,23,24,25,26,27,28,29,30,31,32]. Patient aggression, in particular, elevates stress levels, thereby intensifying all dimensions of burnout [27,29,30]. In this context, the literature increasingly highlights the protective role of personal resources. Psychological resilience, emotional maturity, self-efficacy, adaptive skills, and overall physical and mental well-being serve as buffers against the consequences of chronic occupational stress [19,20]. High levels of these resources facilitate effective coping with professional demands, support emotional regulation, and foster a positive appraisal of the work environment, thereby reducing susceptibility to burnout.

Antonovsky’s theory has been extensively examined in the context of occupational functioning. Research has demonstrated that a high level of sense of coherence positively influences work-related mental well-being and mitigates burnout; such employees show greater engagement, perceive challenges as an inherent component of work, and experience lower stress levels associated with job demands [33,34]. Among psychiatric nurses, a high sense of coherence plays a key role in reducing emotional exhaustion through the activation of personal resources and the utilization of social and professional support [35,36,37,38,39,40]. Coherence fosters the perception of occupational demands as less stressful, significantly lowering the risk of burnout [37,38,39].

Psychiatric nurses with a high sense of coherence exhibit lower levels of emotional exhaustion and depersonalization, coupled with greater job satisfaction [36,37,38,39]. Coherence also serves as a buffer against occupational stress by promoting adaptive coping strategies and limiting the use of maladaptive defense mechanisms [38,40]. A high sense of coherence exerts a protective effect not only against burnout but also against depression and psychological overload. Moreover, it contributes to creating a supportive therapeutic environment, thereby enhancing the effectiveness of psychiatric care [40]. Nurses with strong coherence are more likely to employ adaptive, health-promoting coping strategies oriented toward problem-solving, which facilitates effective tension reduction and minimizes reliance on maladaptive mechanisms that adversely affect mental and physical health [38,40].

An analysis of Polish scientific literature concerning the sense of coherence and occupational burnout among psychiatric nurses indicates significant gaps in empirical research. Existing studies on burnout in this professional group have largely been conducted on limited samples, hindering generalization and cross-study comparison. Although the sense of coherence has been explored in the context of nursing personnel, it has rarely been examined specifically among psychiatric ward staff.

Occupational burnout among nurses has far-reaching implications, extending beyond individual distress to influence patient safety, therapeutic outcomes, and the overall efficiency of healthcare systems. High levels of burnout are associated with increased medical errors, reduced quality of care, higher absenteeism, and elevated turnover rates, which in turn exacerbate staffing shortages and place additional strain on healthcare institutions [18,19]. In psychiatric settings, the consequences are particularly serious: impaired emotional engagement, diminished empathy, and depersonalization can disrupt the therapeutic alliance and negatively affect patient recovery trajectories [11,24].

In Poland, psychiatric nursing is carried out under conditions of persistent staffing shortages, high patient-to-nurse ratios, and limited access to specialized support resources. These systemic challenges may intensify occupational stress and burnout risk, underscoring the importance of examining resilience-related factors, such as the sense of coherence, in this professional group [3,6,23].

The novelty of the present study lies in its focus on the intersection of sense of coherence and burnout within a narrowly defined, high-risk professional group—psychiatric nurses working in Poland. Existing research on burnout in nursing has largely addressed general hospital or mixed healthcare settings, with few studies systematically examining the protective role of sense of coherence in psychiatric wards. By addressing this gap, the study contributes to a deeper understanding of resilience mechanisms in one of the most psychologically demanding nursing specialties.

The aim of the present study was to assess the relationship between the level of sense of coherence and occupational burnout among psychiatric nurses. Our hypothesis was that elevated sense of coherence would associate negatively with emotional exhaustion, depersonalization, and reduced personal accomplishment—the dimensions of occupational burnout.

## 2. Materials and Methods

### 2.1. Study Design and Data Collection

The research was conducted using a cross-sectional design. It incorporated both the estimation method and standardized scaling technique through the use of scale sheets, along with the diagnostic survey method utilizing a questionnaire approach [41]. The selection of research facilities followed a purposive sampling strategy, aiming to include psychiatric wards with varied profiles to enhance the representativeness of the sample and expand the scope of the analysis.

Prior to initiating data collection, formal written permission was obtained from the directors of selected facilities. Requests were submitted to eight psychiatric hospitals located in southern Poland; however, three declined participation. Ultimately, the study was carried out in inpatient psychiatric wards of five hospitals situated in the Lesser Poland and Silesia regions. Data collection commenced in January 2023 and concluded in June 2023.

The participant group was selected purposively, primarily based on the availability of the target population, and comprised individuals working as nurses in inpatient psychiatric settings who met predefined inclusion criteria. These criteria included current employment as a nurse in an inpatient psychiatric ward and voluntary informed consent to participate. Individuals holding non-nursing positions—despite possessing nursing qualifications—or those who did not consent were excluded. A control group was not included in the study design. In this study, the term psychiatric nurses refers to both female and male registered nurses employed in psychiatric inpatient wards, regardless of their specialization or professional title. This includes nurses working across all types of hospital-based psychiatric units. For an international readership, it is important to note that equivalent roles may be described as mental health nurses, behavioural health nurses, or psychiatric–mental health nurses in other countries. The scope of practice, required training, and professional titles for such roles vary substantially across healthcare systems worldwide. In the context of the Polish healthcare system—and specifically for the purposes of this paper—the term encompasses all nurses providing direct care in hospital-based psychiatric wards.

The research procedure consisted of a single phase. Nurses who met the inclusion criteria and provided informed consent received comprehensive information about the aim, scope, and procedures of the study. They were then asked to complete the scale sheets and questionnaires.

All nurses employed in the selected inpatient psychiatric wards were invited to participate in the study, with survey materials distributed proportionally to the size of the nursing staff at each hospital. The overall acceptance rate across sites was approximately 63–73%. A total of 593 nurses agreed to participate; however, only 555 fully completed questionnaires were included in the statistical analyses, as the remaining responses were incomplete and thus excluded. Potential differences between respondents and non-respondents could not be assessed due to the anonymized nature of the data.

### 2.2. Measurement Tools

The study utilized standardized research instruments: The Sense of Coherence Questionnaire (SOC-29), Maslach Burnout Inventory (MBI) and a self-designed questionnaire to collect sociodemographic data.

- The Sense of Coherence Questionnaire (SOC-29), developed by Aaron Antonovsky, was used to assess the level of sense of coherence, defined as a global orientation expressing the extent to which an individual perceives incoming stimuli as comprehensible, manageable, and meaningful. SOC-29 measures the overall index of sense of coherence and consists of three subscales: comprehensibility, manageability, and meaningfulness. The scale comprises 29 items, each rated on a 7-point Likert scale, where the midpoint value of “4” represents a neutral response. Each item provides logically consistent, opposite statements at both ends of the scale. The overall score is calculated by summing the results of the three subscales. The comprehensibility subscale consists of 11 items (1, 3, 5, 10, 12, 15, 17, 19, 21, 24, 26), with scores ranging from 11 to 77 points. Manageability is assessed using 10 items (2, 6, 9, 13, 18, 20, 23, 25, 27, 29), yielding scores from 10 to 70 points. Meaningfulness comprises 8 items (4, 7, 8, 11, 14, 16, 22, 28), with a score range of 8 to 56 points. In the entire SOC-29 questionnaire, the minimum total score is 29 and the maximum is 203. There are no standardized norms; higher total scores indicate a stronger sense of coherence. The same interpretation applies to each subscale: the more points obtained, the more intense the given component. The Polish version of the SOC-29 was developed by the Department of Clinical Psychology of the Institute of Psychiatry and Neurology in Warsaw, the Department of Psychoprophylaxis of the Institute of Psychology at Adam Mickiewicz University in Poznań, and the Department of Occupational Psychology of the Institute of Occupational Medicine in Łódź. The reliability of the Polish version was determined using split-half internal consistency with the Spearman–Brown correction. Cronbach’s α coefficients were 0.68 for meaningfulness, 0.72 for manageability, 0.78 for comprehensibility, and 0.92 for the overall score [42].
- Maslach Burnout Inventory (MBI) developed by Christina Maslach and Susan Jackson, was used to measure occupational burnout across its three dimensions: emotional exhaustion, depersonalization, and personal accomplishment. The tool consists of 22 items, each assigned to one of the three subscales and rated on a 7-point frequency scale (0—“never”; 1—“a few times a year”; 2—“once a month”; 3—“a few times a month”; 4—“once a week”; 5—“a few times a week”; 6—“every day”). The emotional exhaustion subscale comprises 9 items (1, 2, 3, 6, 8, 13, 14, 16, 20) with a maximum score of 54 points. The depersonalization subscale consists of 5 items (5, 10, 11, 15, 22) with a maximum of 30 points. The personal accomplishment subscale includes 8 items (4, 7, 9, 12, 17, 18, 19, 21) with a maximum of 48 points. Higher scores in emotional exhaustion and depersonalization indicate greater burnout, whereas lower scores in personal accomplishment reflect a higher degree of burnout. The internal consistency of the questionnaire has been confirmed, and factor analysis supports its three-factor structure. Cronbach’s α coefficients were established at 0.90 for emotional exhaustion, 0.79 for depersonalization, and 0.71 for personal accomplishment [43].
- The self-designed questionnaire covered items related to gender, age, place of residence, education level, professional specializations, total length of nursing experience, duration of employment in psychiatric wards, type of psychiatric unit currently worked in, current employment fraction, work schedule, managerial responsibilities, marital status, and parenthood. The survey consisted of both closed-ended and semi-open questions.

### 2.3. Data Analysis

Statistical analysis of the collected data was conducted using IBM SPSS Statistics 25 software.

Descriptive statistics were calculated for both dependent and independent variables. For categorical data, frequencies (n) and percentages (%) were reported. In the case of quantitative variables, measures such as the mean (M), standard deviation (SD), median (Me), as well as minimum and maximum values were determined. Additionally, distribution characteristics were assessed using skewness (Sk.) and kurtosis (Kurt).

To assess the normality of the quantitative variables, Kolmogorov–Smirnov tests were conducted. All variables showed deviations from the Gaussian distribution. Therefore, skewness values were further examined. In line with widely accepted guidelines, skewness values within the range of ±2 were interpreted as indicating no substantial asymmetry relative to the mean. This condition was met for all analysed variables. Based on these findings, and with other relevant assumptions fulfilled, parametric tests were applied where appropriate. Relationships between variables were analysed using both parametric and non-parametric methods, including Pearson’s correlation. In addition, linear regression modelling was performed to explore associations between the outcome variable and one or more predictors.

As an additional analysis, the reliability of the research instruments used in this study was evaluated. For the SOC-29 scale, Cronbach’s alpha values were 0.95 for comprehensibility, 0.95 for manageability, 0.96 for meaningfulness, and 0.98 for the overall sense of coherence. For the MBI, Cronbach’s alpha values were 0.95 for emotional exhaustion, 0.91 for depersonalization, and 0.85 for reduced personal accomplishment. These results indicate satisfactory internal consistency for all included subscales.

A threshold of statistical significance was set at *p* < 0.05.

### 2.4. Ethical Considerations

On 12 October 2022, the research project obtained a favorable opinion from the Bioethics Committee of Jagiellonian University (approval number: 1072.6120.213.2022). Respondents were clearly informed that their participation was fully voluntary and anonymous. They were also assured of their right to discontinue completing the questionnaires at any point during the study, without the need to justify their decision and without facing any adverse consequences. No financial or material compensation was offered for taking part in the research. The project was carried out in accordance with the principles of Good Scientific Practice [44] and the Declaration of Helsinki [45].

## 3. Results

### 3.1. Sociodemographic Characteristics of Respondents

A total of 555 completed questionnaires were included in the analysis. The sample consisted of 449 women (80.9%) and 106 men (19.1%). Participants’ ages ranged from 22 to 62 years, with a mean age of 43.07 years (SD = 11.65). Length of employment in psychiatric wards varied from 6 months to 43 years (M = 17.14; SD = 11.76), while overall nursing experience ranged between 1 and 43 years (M = 20.23; SD = 12.39). Additional sociodemographic characteristics of the study group are summarized in Table 1.

### 3.2. Sense of Coherence Level of Psychiatric Nurses

The distribution of global sense of coherence scores among nurses employed in inpatient psychiatric wards ranged from 30 to 184 points. The mean score was 124.67 (SD = 45.81). The results for the individual components of the sense of coherence are presented in Table 2.

### 3.3. Level of Occupational Burnout Among Psychiatric Nurses

The mean score for emotional exhaustion in the studied group was 28.75 (SD = 16.39), for depersonalization 13.55 (SD = 9.71), and for reduced personal accomplishment 23.61 (SD = 11.11). Detailed results are presented in Table 3.

### 3.4. The Relationship Between Sense of Coherence and Occupational Burnout Levels in a Group of Psychiatric Nurses

The analysis revealed a significant negative correlation between the overall sense of coherence, including its three components, and the level of occupational burnout among nurses working in inpatient psychiatric wards. Higher scores in comprehensibility, manageability, and meaningfulness were associated with lower levels of emotional exhaustion, depersonalization, and reduced personal accomplishment (*p* < 0.001) (Table 4).

The correlation analysis revealed a significant negative relationship between the overall sense of coherence, its three components, and the level of emotional exhaustion among nurses working in inpatient psychiatric wards. Participants with higher levels of comprehensibility, manageability, and meaningfulness reported lower levels of emotional exhaustion (*p* < 0.001). A linear regression analysis, with emotional exhaustion as the dependent variable and the three components of sense of coherence as predictors, yielded a statistically significant model (F(3, 551) = 40.52; *p* < 0.001; R^2^ = 0.18). Among the predictors, manageability was found to be a significant factor (β = −0.73; *p* < 0.001), indicating that individuals with higher levels of this resource experienced lower emotional exhaustion (Table 5).

The study also found significant negative correlations between the sense of coherence (including all components) and the level of depersonalization. Higher levels of manageability and meaningfulness were associated with lower levels of depersonalization (*p* < 0.001 and *p* = 0.012, respectively). The regression model for depersonalization was statistically significant (F(3, 551) = 19.16; *p* < 0.001; R^2^ = 0.09). Manageability (β = −0.65; *p* < 0.001) and meaningfulness (β = 0.37; *p* = 0.012) emerged as significant predictors, suggesting that individuals with stronger resources in these areas experienced less depersonalization (Table 6).

Negative correlations were also observed between sense of coherence (global and its components) and reduced personal accomplishment. The strongest inverse relationship was found for manageability (*p* < 0.001). The linear regression model predicting reduced personal accomplishment was statistically significant (F(3, 551) = 90.19; *p* < 0.001; R^2^ = 0.33). Manageability was identified as the only significant predictor (β = −0.65; *p* < 0.001), indicating that higher levels of this component were associated with a lower sense of reduced personal accomplishment (Table 7).

## 4. Discussion

Previous scientific analyses suggest that the assessment of the sense of coherence among nurses working in psychiatric wards has been addressed relatively infrequently in both national and international research. Despite the specific working conditions in psychiatric care—including distinct requirements, a high intensity of interpersonal contact, and significant emotional burden—most existing publications have focused on nursing staff employed in other areas of clinical care [40,46,47]. This research gap highlights the need for in-depth studies in this field to better understand the mechanisms that support the maintenance of mental well-being among psychiatric staff.

The analysis of the present study revealed that both the overall level of sense of coherence and its individual components were at an average level. In the study conducted by Berg & Hallberg, the impact of supportive interventions for nursing staff in Swedish psychiatric wards was examined, with particular attention to changes in the sense of coherence. The intervention program included regular clinical supervision for staff and the implementation of structured, monitored patient care. Clinical supervision involved periodic meetings between nurses and experienced supervisors, during which professional issues were discussed, strategies for coping with emotional strain and workplace conflicts were developed, and reflective practices were encouraged. Supervised care focused on the systematic documentation of the therapeutic process and the adjustment of nursing interventions to the individual needs of patients. The study included 16 female nurses and 6 male nurses. The mean sense of coherence score prior to the program was 146.6 points (SD = 21.2), which increased to 153.6 points (SD = 18.3) after one year of the intervention. These results indicate that the implemented support measures contributed to an improvement in the sense of coherence, which was already relatively high at the beginning of the study. By enhancing competencies in coping with occupational stress, clinical supervision promoted the perception of work as more structured, predictable, and meaningful, which may have indirectly influenced the development of psychological resilience and professional effectiveness [46].

Similarly, Van der Colff & Rothmann, in a study of 818 South African nurses—including psychiatric staff—reported an average sense of coherence score of 137.95 points (SD = 20.46). Differences between the obtained results may be attributed to sample size, the specific functioning of psychiatric wards in different countries, and varying levels of access to social and organizational support networks [48].

The sense of coherence, as described in Aaron Antonovsky’s salutogenic model, plays a role in interpreting demanding professional experiences by making them appear more predictable and structured. This mode of perception facilitates more effective coping with stress and emotional demands at work [2]. The findings of Berg & Hallberg [46] indicate that systematic implementation of interventions aimed at developing coping strategies, analyzing emotionally challenging situations, and planning individualized patient care can significantly enhance the sense of coherence while simultaneously reducing the risk of burnout. This has important implications for clinical practice, emphasizing the need to implement programs that strengthen coherence among staff. Such initiatives may not only enhance nurses’ psychological resilience but also contribute to greater job satisfaction.

A review of the literature indicates that the issue of burnout among psychiatric nurses has been relatively frequently addressed in international publications. Many of these studies focus both on identifying factors contributing to burnout in this professional group and on developing strategies to mitigate its effects—at both the individual and organizational levels [19,21,49]. A challenge in comparing findings arises from the interpretation of the personal accomplishment subscale. Some authors present the results according to the original scoring method, where higher values indicate lower burnout levels. Others reverse the scoring, adopting the perspective of reduced personal accomplishment, in which higher values indicate higher burnout levels, thus ensuring consistency with the other subscales. These differences in presentation require particular caution when interpreting results.

The findings indicate a moderate-to-high level of occupational burnout among the participants, with scores in all three MBI subscales suggesting the presence of emotional exhaustion, depersonalization, and reduced personal accomplishment. In the study by Konstantinou et al., involving 78 Greek psychiatric nurses, the mean emotional exhaustion score was 26.90 points (SD = 13.9), depersonalization—8.04 points (SD = 6.86), and reduced personal accomplishment—10.68 points (SD = 8.87) [50]. In contrast, Tang et al., examining the relationship between organizational support and burnout among 916 Chinese psychiatric nurses, reported mean values of 24.16 points (SD = 9.98) for emotional exhaustion, 10.27 points (SD = 5.07) for depersonalization, and 19.28 points (SD = 7.26) for personal accomplishment [19]. In the study by Suazo Galdames et al., conducted among 1165 Spanish nurses and exploring the relationship between resilience and burnout, the mean emotional exhaustion score was 29.76 points (SD = 12.33), depersonalization—9.56 points (SD = 6.04), and personal accomplishment—37.57 points (SD = 6.27) [51]. A comparison of these findings indicates that the level of burnout among psychiatric nurses across the studied populations remains moderate in all three dimensions, despite some differences between individual studies.

Occupational burnout among psychiatric nurses can be mitigated by implementing interventions targeting both individual support and organizational change, or by combining both approaches [49,52,53]. Alenezi, McAndrew & Fallon conducted an experimental study assessing the effectiveness of a burnout prevention program in this professional group. The study involved 296 nurses, with 158 assigned to the intervention group and 142 to the control group. Measurements taken 1, 3, and 6 months after the completion of the program confirmed a reduction in burnout levels among the intervention participants. The program consisted of two-day workshops covering modules on stress management, relaxation techniques, social skills training, and effective communication. Although a gradual increase in burnout indicators was observed after six months, they did not return to baseline levels, suggesting that the intervention produced long-lasting positive effects [52]. The beneficial impact of Mindfulness self-help programs on reducing burnout and enhancing resilience in this professional group has also been confirmed [53].

In the present study, a significant relationship was found between the overall sense of coherence, as well as its three components, and the severity of occupational burnout among respondents. Participants with higher levels of comprehensibility, manageability, meaningfulness, and total sense of coherence exhibited lower levels of emotional exhaustion, depersonalization, and reduced personal accomplishment. Regression analysis revealed that manageability was a significant predictor of all burnout dimensions, while meaningfulness additionally influenced depersonalization. These findings are consistent with the literature: Van der Colff & Rothmann observed that a high sense of coherence among psychiatric nurses was associated with lower burnout levels across all three dimensions [48]. Similar conclusions were drawn by Xue et al. in a study of 302 nurses, which demonstrated an inverse relationship between sense of coherence and burnout symptoms [54]. Likewise, Pachi et al., in a study involving 660 nurses, confirmed that lower sense of coherence correlated with higher levels of occupational burnout [55]. Although comprehensibility and meaningfulness did not significantly predict certain dimensions of burnout, these components remain theoretically relevant within Antonovsky’s model. Their non-significant predictive value in this study may reflect contextual factors, such as organizational support or cultural differences, which could moderate the relationship between sense of coherence and burnout. Future research should explore these potential moderators to better understand the complex interplay between coherence components and occupational outcomes. Cross-cultural differences in nursing practice and organizational support may influence both sense of coherence and burnout levels. For instance, access to clinical supervision, staffing ratios, and cultural norms regarding emotional expression could shape how coherence impacts occupational stress, highlighting the need for context-sensitive interventions across different healthcare systems.

This study should be assessed in light of its strengths and limitations. A major strength lies in its detailed examination of sense of coherence and its relationship with burnout levels among psychiatric nurses, a topic that has been seldom explored in Polish scientific literature. The solid theoretical basis of the study ensures its alignment with existing research, thereby increasing the reliability and validity of the results. The use of standardized research instruments enables replication in multicenter settings and with randomized sampling, which allows for broader comparisons within the population of psychiatric nurses. Among the study’s limitations are its cross-sectional design, which limits the ability to draw causal conclusions. The purposive sampling method, relatively small sample size, and restriction of the research area to selected regions of Poland may limit the generalizability of the findings. A potential limitation is also the response bias due to the self-report nature of the SOC-29 and MBI instruments, which may have influenced participants’ responses. Therefore, caution is necessary when extrapolating the results to the wider population of psychiatric nurses.

### Implication for Practice

Sense of coherence can act as a protective factor in work settings that are characterized by intense emotional and cognitive demands, supporting the ability to maintain mental stability and professional efficiency. Regular evaluation of coherence levels alongside occupational stress among psychiatric nursing staff allows for the early detection of individuals at risk of burnout and facilitates the design of targeted support strategies. Such assessments can generate valuable insights for developing preventive initiatives and structured programs that strengthen adaptive coping mechanisms at both the personal and institutional level. Promoting a strong sense of coherence within nursing teams may enhance their capacity to perceive work-related challenges as manageable and meaningful, thereby improving overall well-being and the quality of patient care.

The results of the current study indicate that fostering a high sense of coherence among nurses working in psychiatric inpatient units holds considerable practical importance. Preventing chronic stress and emotional exhaustion in this professional group requires deliberate reinforcement of personal resources, with coherence serving as a key moderating factor in stress management. As an essential element of professional competencies, a well-developed sense of coherence enables more effective functioning under pressure, offers a psychological safeguard in demanding clinical environments, and contributes to maintaining safe and consistent patient care. Therefore, it becomes vital to create and implement specialized training modules and workshops designed to strengthen coherence as part of postgraduate education and continuous in-house development programs, ideally conducted under the supervision of experienced mentors.

Additionally, practical interventions could include structured clinical supervision models, resilience training programs, and mindfulness-based stress reduction sessions. Clinical supervision allows nurses to reflect on emotionally challenging cases, enhancing predictability and perceived control in daily work. Resilience training, encompassing cognitive-behavioral techniques and emotional regulation skills, may strengthen the sense of manageability and meaningfulness, equipping nurses with adaptive coping strategies. Mindfulness programs increase awareness of emotional reactions and reduce emotional exhaustion, reinforcing the protective role of coherence. Organizational interventions, such as balanced staffing ratios, flexible scheduling, and access to peer support or mentoring, further support coherence by creating a work environment that nurses can understand, manage, and find meaningful. Implementing these measures alongside regular assessment of sense of coherence may maximize their effectiveness and contribute to staff well-being and high-quality patient care.

## 5. Conclusions

The interpretation of the obtained results allows for several key conclusions. The overall sense of coherence among nurses working in inpatient psychiatric wards, as well as its components—comprehensibility, manageability, and meaningfulness—can be classified as moderate. The intensity of occupational burnout symptoms in the studied group was also moderate, with emotional exhaustion being the most prominent dimension, followed by depersonalization and reduced personal accomplishment. A higher level of overall sense of coherence was significantly associated with lower levels of occupational burnout across all dimensions, with manageability emerging as the strongest predictor of reduced emotional exhaustion, depersonalization, and diminished personal accomplishment.

Regular assessment of the sense of coherence among psychiatric nursing staff may facilitate early identification of individuals at heightened risk of burnout. Incorporating such assessments into institutional mental health strategies can support the development of effective, evidence-based psychological and organizational interventions aimed at improving nurses’ well-being and enhancing the quality of psychiatric care.

Furthermore, future research could benefit from longitudinal or intervention-based studies to examine causal mechanisms and changes in sense of coherence over time, providing deeper insights into strategies for preventing burnout among psychiatric nurses.

## Figures and Tables

**Table 1:** Sociodemographic characteristics of respondents.

		n	%
Place of residence	Village	210	37.8
	City < 250 thousand inhabitants	172	31.0
	City > 250 thousand inhabitants	173	31.1
Level of education	Secondary vocational education	163	29.4
	Bachelor’s degree	147	26.5
	Master’s degree	236	42.5
	Doctoral degree/academic title	9	1.6
Specialization in the field of nursing	No specialization	202	36.4
	In progress	99	17.8
	Completed (including seven individuals currently pursuing a second specialization)	254	45.8
Work-time ratio in psychiatric wards	0.25	4	0.7
	0.5	41	7.5
	0.75	5	0.9
	1	489	88.0
	1.25	1	0.2
	1.5	13	2.3
	2	2	0.4
Work-time ratio in other wards	0	380	68.5
	0.25	15	2.7
	0.33	4	0.7
	0.5	101	18.2
	0.75	8	1.4
	1	42	7.6
	1.5	5	0.9
Type of psychiatric ward	General psychiatric ward	130	23.4
	Child and adolescent psychiatry ward	29	5.2
	Basic security forensic psychiatry ward	61	11.0
	Enhanced security forensic psychiatry ward	64	11.5
	Enhanced security forensic psychiatry ward for juveniles	12	2.2
	Psychiatric rehabilitation ward	40	7.2
	Psychogeriatric ward	46	8.3
	Alcohol detoxification ward	40	7.2
	Alcohol addiction treatment ward	58	10.5
	Psychoactive substance addiction treatment ward	18	3.2
	Personality and neurotic disorders treatment ward	13	2.3
	General psychiatry ward with basic security forensic psychiatry subunit	18	3.2
	Long-term psychiatric care ward	19	3.4
	More than one ward	7	1.3
Work arrangement	Day shift	81	14.6
	Rotating shifts	474	85.4
Management position	Yes	58	10.5
	No	497	89.5
Marital status	Single	114	20.6
	Informal relationship	75	13.6
	Married	278	50
	Divorced	60	10.8
	Widowed	28	5.0
Parental status	Yes	386	69.6
	No	169	30.4
Number of children	1	141	25.4
	2	154	27.7
	3	57	10.3
	4	21	3.8
	5	6	1.1
	6	3	0.5

n—number of individuals; %—percentage.

**Table 2:** Sense of coherence level of psychiatric nurses.

Sense of Coherence—The Sense of Coherence Questionnaire (SOC-29)	M	Me	SD	Sk.	Kurt.	Min	Max	D	*p*
Global sense of coherence	124.68	133	45.81	−0.69	−0.63	30	184	0.12	<0.001
Comprehensibility	42.70	44	15.83	−0.57	−0.76	12	69	0.12	<0.001
Manageability	43.83	47	16.28	−0.68	−0.66	10	68	0.14	<0.001
Meaningfulness	38.16	42	14.65	−0.68	−0.68	8	56	0.11	<0.001

M—mean; Me—median; SD—standard deviation; Sk.—skewness; Kurt.—kurtosis; Min—minimum value; Max—maximum value; D—Kolmogorov–Smirnov test statistic; *p*—significance level of the Kolmogorov–Smirnov test.

**Table 3:** Level of occupational burnout among psychiatric nurses.

Occupational Burnout—Maslach Burnout Inventory (MBI)	M	Me	SD	Sk.	Kurt.	Min	Max	D	*p*
Emotional exhaustion	28.75	26	16.39	0.05	−1.45	1	54	0.16	<0.001
Depersonalization	13.55	11	9.71	0.27	−1.47	0	30	0.16	<0.001
Reduced personal accomplishment	23.61	24	11.11	0	−0.73	1	47	0.08	<0.001

M—mean; Me—median; SD—standard deviation; Sk.—skewness; Kurt.—kurtosis; Min—minimum value; Max—maximum value; D—Kolmogorov–Smirnov test statistic; *p*—significance level of the Kolmogorov–Smirnov test.

**Table 4:** The relationship between the level of resilience and the intensity of perceived stress among the respondents.

Sense of Coherence—The Sense of Coherence Questionnaire (SOC-29)	Occupational Burnout—Maslach Burnout Inventory (MBI)					
	Emotional Exhaustion		Depersonalization		Reduced Personal Accomplishment	
	r	*p*	r	*p*	r	*p*
Global sense of coherence	−0.39	<0.001	−0.27	<0.001	−0.56	<0.001
Comprehensibility	−0.37	<0.001	−0.25	<0.001	−0.52	<0.001
Manageability	−0.42	<0.001	−0.29	<0.001	−0.57	<0.001
Meaningfulness	−0.37	<0.001	−0.24	<0.001	−0.54	<0.001

r—Pearson’s correlation coefficient; *p*—significance level.

**Table 5:** Regression model for emotional exhaustion with sense of coherence dimensions as predictors.

Sense of Coherence—The Sense of Coherence Questionnaire (SOC-29)	Dependent Variable—Emotional Exhaustion—Maslach Burnout Inventory (MBI)				
	*B*	*SE*	*β*	*t*	*p*
Constant	46.65	1.84		25.31	<0.001
Comprehensibility	0.12	0.12	0.11	0.96	0.337
Manageability	−0.73	0.14	−0.73	−5.09	<0.001
Meaningfulness	0.24	0.16	0.22	1.58	0.116

*B*—unstandardized coefficient; *SE*—standard error; *β*—standardized coefficient; *t*—*t*-test value; *p*—significance level.

**Table 6:** Regression model for depersonalization with sense of coherence dimensions as predictors.

Sense of Coherence—The Sense of Coherence Questionnaire (SOC-29)	Dependent Variable—Depersonalization—Maslach Burnout Inventory (MBI)				
	*B*	*SE*	*β*	*t*	*p*
Constant	20.88	1.15		18.18	<0.001
Comprehensibility	0.01	0.08	0.02	0.12	0.903
Manageability	−0.39	0.09	−0.65	−4.33	<0.001
Meaningfulness	0.25	0.10	0.37	2.53	0.012

*B*—unstandardized coefficient; *SE*—standard error; *β*—standardized coefficient; *t*—*t*-test value; *p*—significance level.

**Table 7:** Regression model for reduced personal accomplishment with sense of coherence dimensions as predictors.

Sense of Coherence—The Sense of Coherence Questionnaire (SOC-29)	Dependent Variable—Reduced Personal Accomplishment—Maslach Burnout Inventory (MBI)				
	*B*	*SE*	*β*	*t*	*p*
Constant	40.53	1.13		35.86	<0.001
Comprehensibility	0.08	0.07	0.11	1.01	0.314
Manageability	−0.45	0.09	−0.65	−5.03	<0.001
Meaningfulness	−0.02	0.10	−0.02	−0.17	0.864

*B*—unstandardized coefficient; *SE*—standard error; *β*—standardized coefficient; *t*—*t*-test value; *p*—significance level.

## Data Availability

Data are available from the project manager.

## Public Involvement Statement

No public involvement in any aspect of this research.

## Guidelines and Standards Statement

This manuscript was drafted against the STROBE (Strengthening the Reporting of Observational Studies in Epidemiology) guidelines for cross-sectional studies.

## Abbreviations

The following abbreviations are used in this manuscript:

F	F-statistic value
MBI	Maslach Burnout Inventory
R^2^	coefficient of determination
SOC-29	The Sense of Coherence Questionnaire
